# Is dynapenic abdominal obesity a risk factor for cardiovascular mortality? A competing risk analysis

**DOI:** 10.1093/ageing/afac301

**Published:** 2023-01-09

**Authors:** Paula Camila Ramírez, Dayane Capra de Oliveira, Roberta de Oliveira Máximo, Aline Fernanda de Souza, Mariane Marques Luiz, Maicon Luís Bicigo Delinocente, Andrew Steptoe, Cesar de Oliveira, Tiago da Silva Alexandre

**Affiliations:** Programa de Pós-Graduação em Fisioterapia, Universidade Federal de São Carlos, São Carlos, Brazil; Escuela de Fisioterapia, Universidad Industrial de Santander, Bucaramanga, Colombia; Programa de Pós-Graduação em Fisioterapia, Universidade Federal de São Carlos, São Carlos, Brazil; Programa de Pós-Graduação em Fisioterapia, Universidade Federal de São Carlos, São Carlos, Brazil; Programa de Pós-Graduação em Fisioterapia, Universidade Federal de São Carlos, São Carlos, Brazil; Programa de Pós-Graduação em Fisioterapia, Universidade Federal de São Carlos, São Carlos, Brazil; Programa de Pós-Graduação em Gerontologia, Universidade Federal de São Carlos, São Carlos, Brazil; Department of Epidemiology & Public Health, University College London, London, UK; Department of Epidemiology & Public Health, University College London, London, UK; Programa de Pós-Graduação em Fisioterapia, Universidade Federal de São Carlos, São Carlos, Brazil; Programa de Pós-Graduação em Gerontologia, Universidade Federal de São Carlos, São Carlos, Brazil; Department of Epidemiology & Public Health, University College London, London, UK; Departamento de Gerontologia, Universidade Federal de São Carlos, São Carlos, Brazil

**Keywords:** dynapenia, abdominal obesity, mortality, cardiovascular disease, English Longitudinal Study of Ageing (ELSA) study, older people

## Abstract

**Background:**

Dynapenic abdominal obesity has been shown as a risk factor for all-cause mortality in older adults. However, there is no evidence on the association between this condition and cardiovascular mortality.

**Objective:**

We aimed to investigate whether dynapenic abdominal obesity is associated with cardiovascular mortality in individuals aged 50 and older.

**Methods:**

A longitudinal study with an 8-year follow-up was conducted involving 7,030 participants of the English Longitudinal Study of Ageing study. Abdominal obesity and dynapenia were respectively defined based on waist circumference (> 102 cm for men and > 88 cm for women) and grip strength (< 26 kg for men and < 16 kg for women). The sample was divided into four groups: non-dynapenic/non-abdominal obesity (ND/NAO), non-dynapenic/abdominal obesity (ND/AO), dynapenic/non-abdominal obesity (D/NAO) and dynapenic/abdominal obesity (D/AO). The outcome was cardiovascular mortality. The Fine-Grey regression model was used to estimate the risk of cardiovascular mortality as a function of abdominal obesity and dynapenia status in the presence of competing events controlled by socio-demographic, behavioural and clinical variables.

**Results:**

The risk of cardiovascular mortality was significantly higher in individuals with D/AO compared with ND/NAO (SHR 1.85; 95% CI: 1.15–2.97). D/NAO was also associated with cardiovascular mortality (SHR: 1.62; 95% CI: 1.08–2.44).

**Conclusion:**

Dynapenic abdominal obesity is associated with cardiovascular mortality, with a larger effect size compared to dynapenia alone in individuals older than 50 years. Thus, prevention strategies and clinical interventions that enable mitigating the harmful effects of these conditions should be adopted to diminish such risk.

## Key Points

- Dynapenic abdominal obesity and dynapenia are associated with cardiovascular mortality.
- Obesity alone was not associated with cardiovascular mortality.
- The combined physiopathological effect of abdominal obesity and dynapenia increases the risk of cardiovascular mortality.

## Introduction

Cardiovascular disease is the major cause of death. In 2019, the World Health Organization reported that ischemic heart disease and stroke, respectively, accounted for 16 and 11% of all deaths in the world [1–3]. The prevalence of these diseases and their consequences increase with the advance in age and are related to changes in body composition occurring throughout life [4].

Ageing is associated with significant changes in body composition, with a substantial loss of the muscle mass and the accumulation of abdominal fat, giving rise to the phenotype known as sarcopenic obesity (combination of sarcopenia and obesity). Sarcopenic obesity has been associated with an increased risk of cardiovascular disease in some longitudinal studies [5] but not in others [6]. The most marked change in ageing is related to a gradual decline in neuromuscular strength known as dynapenia, which is mediated by physiological neuromuscular adaptations, such as the loss of alpha motor neurons, deficiencies in neural activation and motor recruitment patterns, the loss of type II fibres and the predominance of type I fibres, accompanied by the accumulation of intramuscular fat [7, 8]. Since its introduction, the novel concept of dynapenic abdominal obesity [9] has been linked to adverse outcomes, such as disability regarding instrumental and basic activities of daily living, falls, slow gait speed, worse physical performance and hospitalisation in older adults [10–17]. It has also been considered to be more important than sarcopenic obesity regarding the risk of cardiovascular disease in old age [5].

Studies have reported conflicting findings on the association between dynapenic abdominal obesity and all-cause mortality and none has evaluated the possible association with mortality due to cardiovascular disease. For instance, in a study involving 6,173 participants of the English Longitudinal Study of Ageing (ELSA) and the Brazilian Health, Well-being and Ageing Study (SABE) with a 10-year follow-up, Alexandre and colleagues [18] found a greater risk of mortality among participants with dynapenic abdominal obesity. Rossi and colleagues [16] found a similar result in a study involving 262 Italian physicians, which also had a 10-year follow-up period. In a subsequent study involving 846 participants of the *Invecchiare nel Chianti* (InCHIANTI) study in Italy with an 11-year follow-up; however, Rossi and colleagues [17] found that the association between dynapenic abdominal obesity and mortality was non-significant.

The simultaneous occurrence of abdominal obesity and dynapenia in older adults has systemic repercussions that increase cardiovascular risk by diminishing anabolic pathways as well as increasing proinflammatory activity, insulin resistance and oxidative stress due to the synergic action of the two conditions [19, 20]. These mechanisms impair lipid and carbohydrate metabolism, the renin-angiotensin system and sympathetic activity, alter muscle contractibility with repercussions for cardiomyocytes and generate endothelial dysfunction and atherosclerosis, which increases the risk of developing cardiovascular disease and, consequently, the risk of death [21–26]. Therefore, the present study aimed to investigate whether dynapenic abdominal obesity is associated with cardiovascular mortality in individuals aged 50 and older participating in the ELSA study.

## Methods

### Study population

ELSA is a panel study initiated in 2002 in the UK with a nationally representative sample of community-dwelling individuals 50 years of age or older. ELSA interviews occur every 2 years with the administration of questionnaires. Health examinations, blood collection for the determination of biochemical measures and performance tests occur every 4 years with the visit of a nurse to the participants’ homes. The present analysis included 7,030 individuals aged 50 and older who participated in Wave 2 (2004/5). We examined all cardiovascular deaths occurred during the 8-year follow-up (2012/3). Detailed information about sample selection and a flowchart can be found in the Supplemental Material (Section study population).

### Cardiovascular mortality

Information on dates of death and respective causes were obtained from the UK National Health Service Mortality Registry. Detailed information can be found in the Supplemental Material.

### Dynapenic abdominal obesity

Abdominal obesity was determined based on waist circumference > 102 cm for men and > 88 cm for women [27]. Muscle strength was determined based on the grip strength. Dynapenia was defined as grip strength <26 kg for men and < 16 kg for women [28]. Detailed information can be found in the Supplemental Material.

ELSA participants were divided into four groups based on their dynapenia and abdominal obesity status: non-dynapenic/non-abdominal obesity (ND/NAO), non-dynapenic/abdominal obesity (ND/AO), dynapenic/non-abdominal obesity (D/NAO) and dynapenic/abdominal obesity (D/AO) [29].

### Covariates

The covariates included in the present analysis constitute a broad spectrum of factors associated with mortality [16,30,31], such as sex, age, marital status, wealth, schooling, smoking, alcohol intake, physical activity, systemic arterial hypertension, diabetes, cancer, lung disease, heart disease, stroke, depression and body mass index (BMI). Detailed information can be found in the Supplemental Material.

### Ethical considerations

All participants of ELSA signed a statement of informed consent and all waves of the study received approval from the London Multicentre Research Ethics Committee [MREC/01/2/91]).

### Statistical analysis

The sample characteristics were expressed as the mean, standard deviation and proportion. Differences among the four groups based on abdominal obesity and dynapenia status were evaluated using the chi-square test, ANOVA and the Bonferroni post-hoc test. A *P*-value <0.05 was considered indicative of statistical significance.

The Fine-Gray competing-risk model was used for the analysis of the association between dynapenic abdominal obesity and cardiovascular mortality. As the Cox model assumes that censured observations have the same probability of undergoing the event as those that remain in observation and that this presupposition is not met when a person develops a competitive event, such as different causes of death, different approaches have been developed to avoid the overestimation of Cox models [32]. One of the alternatives is the Fine-Gray model, which calculates the rates of events in time *t* for those individuals who presented the event of interest (e.g. death by cardiovascular disease) or could present a competitive event (death by other causes). Thus, the cumulative incidence competing risk is calculated based on the function of cumulative incidence, which is estimated for both the event of interest and competing events, with each estimate depending on the other. In the estimate of parameters, the model attributes time-dependent weights to individuals who continue in the risk group [30]. Finally, the Fine-Gray model produces estimates of the sub-distribution hazard ratio (SHR), which is the instantaneous change in the rate of occurrence of an event among individuals who are free of the outcome or presented some competing event and has demonstrated better predictions of cardiovascular risk in older adults compared to the Cox model [31].

In the models, the ND/NAO group was considered the reference category. Control variables were selected by the theoretical framework including those possible confounding variables of the tested associations; subsequently, those with a *P*-value <0.20 in the bivariate analyses were incorporated into the multiple models, and those with a *P*-value <0.05 were maintained in the final model [33, 34]. Six sensitivity analyses were performed: First, excluding individuals younger than 60 years of age, in order to investigate whether the exclusion of younger participants would be able to modify the associations obtained; second, excluding current smokers; third, excluding individuals with heart disease, cancer, or stroke at baseline because both smokers and participants with heart disease, cancer or stroke may have a higher risk of mortality; fourth, excluding participants less than 60 years of age, smokers and individuals with heart disease, cancer, or stroke at baseline; fifth, using BMI ≥ 30 kg/m^2^ rather than waist circumference to define dynapenic obesity; and sixth, using dynapenia alone (yes/no) and abdominal obesity alone (yes/no) to explore whether using BMI or obesity and dynapenia alone would be capable of modifying the associations found in the original model. All analyses were conducted using STATA 15.0 SE (StataCorp, College Station, TX, USA).

## Results

During the 8-year follow-up, 1,058 participants died, 322 (30.4%) of whom died due to cardiovascular disease. The prevalence of dynapenic abdominal obesity, dynapenia and obesity were 3.8, 3.9 and 47.4%, respectively.

The mean age of the participants was 66.1 ± 9.3 years. Women, individuals with a conjugal life, those with low schooling, those with low physical activity level, those with frequent alcohol intake and ex-smokers predominated in the sample. The most prevalent disease was hypertension, followed by heart disease and lung disease. The majority of the participants had overweight and obesity. The sociodemographic, clinical, behavioural and anthropometric characteristics of the participants at baseline are displayed in Tables 1 and 2.

**Table 1:** Baseline sociodemographic and behavioural characteristics according to abdominal obesity and dynapenia status in 7,030 participants of ELSA (2004/2005)

	Total *n* = 7,030	ND/NAO *n* = 3,159	ND/AO *n* = 3,332	D/NAO *n* = 270	D/AO *n* = 269
**Sociodemographic**					
Sex (female) (%)	54.7	48.7	58.6^a^	61.8^a^	69.9^a,b^
Age, years (SD)	66.1 ± 9.3	65.0 ± 8.9	65.6 ± 8.8^a^	76.6 ± 9.9^a,b^	73.4 ± 10.1^a,b,c^
Age (%)					
50–59	31.4	33.9	32.3	9.6^a,b^	10.8^a,b^
60–69	34.2	36.7	34.3	13.3^a,b^	24.9^a,b^
70 or more	34.4	29.4	33.4^a^	77.1^a,b^	64.3^a,b^
Marital status (with conjugal life) (%)	70.2	73.1	71.6	45.9^a,b^	44.2^a,b^
Total household wealth (%)					
Lowest quintile	15.7	12.0	16.6^a^	31.8^a,b^	33.1^a,b^
2nd quintile	18.7	16.1	20.1^a^	20.4^a^	29.0^a,b^
3rd quintile	20.3	19.7	21.5	17.0	16.4
4th quintile	21.3	23.3	20.2^a^	17.4^a^	15.2^a^
Highest quintile	22.6	27.6	20.1^a^	13.0^a,b^	5.6^a,b,c^
Not applicable	1.4	1.3	1.5	0.4	0.7
Schooling (%)					
0–11 years	50.3	43.4	53.1^a^	74.1^a,b^	73.2^a,b^
12–13 years	24.7	26.4	24.5	14.1^a,b^	16.7^a,b^
> 13 years	25.0	30.2	22.4^a^	11.8^a,b^	10.1ª^,b^
**Behavioural**					
Physical activity (%)					
Inactive	4.2	2.7	3.7^a^	14.4^a,b^	16.7^a,b^
Low	93.4	95.5	93.7^a^	79.3^a,b^	79.9^a,b^
Moderate/vigorous	2.4	1.8	2.6	6.3^a,b^	3.4
Alcohol intake (%)					
Never/rarely	16.8	13.7	18.2^a^	25.9^a,b^	27.5^a,b^
Frequently	41.1	40.1	42.8	36.3	36.1^b^
Daily	32.6	38.4	29.6^a^	17.4^a,b^	16.3^a,b^
Not declared	9.5	7.8	9.4	20.4^a,b^	20.1^a,b^
Smoking status (%)					
Non-smoker	37.0	39.0	35.6^a^	35.6	32.4
Ex-smoker	48.6	45.5	51.0^a^	48.9	56.1^a^
Smoker	14.4	15.5	13.4	15.5	11.5

**Note:** Data expressed as the proportion, mean and standard deviation. Abbreviations: SD = standard deviation. ND/NAO: non-dynapenic/non-abdominally obese. ND/AO: non-dynapenic/abdominally obese. D/NAO: dynapenic/non-abdominally obese; D/AO: dynapenic/abdominally obese. ^a^Significantly different from non-dynapenic/non-abdominally obese; ^b^significantly different from non-dynapenic/abdominally obese; ^c^significantly different from dynapenic/non-abdominally obese (*P* < 0.05).

**Table 2:** Baseline clinical and anthropometric characteristics according to abdominal obesity and dynapenia status in 7,030 participants of English longitudinal study of ageing (2004/5)

	Total *n* = 7,030	ND/NAO *n* = 3,159	ND/AO *n* = 3,332	D/NAO *n* = 270	D/AO *n* = 269
**Clinical conditions** (yes) (%)					
Systemic arterial hypertension	42.8	33.0	50.8^a^	46.3^a^	55.4^a,b^
Diabetes	8.1	4.6	10.8^a^	8.1^a^	16.7^a,b,c^
Cancer	7.6	6.9	8.3	8.5	7.8
Lung disease	17.8	15.9	18.6^a^	19.3	26.8^a,b^
Heart disease	21.6	19.5	21.8	31.5^a,b^	35.3^a,b^
Stoke	4.2	3.6	3.8	11.1^a,b^	9.7^a,b^
Depressive symptoms	14.1	11.4	14.7^a^	26.3^a,b^	26.3^a,b^
**Anthropometry**					
Grip strength, kg (SD)	31.7 ± 11.5	33.6 ± 10.5	32.7 ± 10.9^a^	15.3 ± 5.4^a,b^	14.8 ± 5.3^a,b^
Waist circumference, cm (SD)	95.5 ± 13.0	86.8 ± 9.1	104.1 ± 10.3^a^	84.8 ± 8.6^a,b^	102.7 ± 9.4^a,c^
Body mass index (%)					
Ideal range	27.1	51.1	3.0^a^	63.3^a,b^	7.1^a,b,c^
Underweight	0.8	1.7	0.0	3.0	0.0
Overweight	43.4	45.3	42.7	32.2^a,b^	40.1^b^
Obesity	28.7	1.9	54.3^a^	1.5	52.8^a,c^

**Note:** Data expressed as the proportion, mean and standard deviation. Abbreviations: SD = standard deviation. ND/NAO: non-dynapenic/non-abdominally obese. ND/AO: non-dynapenic/abdominally obese. D/NAO: dynapenic/non-abdominally obese; D/AO: dynapenic/abdominally obese. ^a^Significantly different from non-dynapenic/non-abdominally obese; ^b^significantly different from non-dynapenic/abdominally obese; ^c^significantly different from dynapenic/non-abdominally obese (*P* < 0.05).

The individuals in the D/AO group were predominantly women, older, not married, had lower income and school, were more likely to be physically inactive, consumed less alcohol, were more likely to be ex-smokers, had higher frequencies of heart disease, lung disease, depressive symptoms, hypertension, diabetes and obesity, had a higher mean waist circumference and had a lower mean grip strength compared to those in the ND/NAO group. In comparison with the ND/AO group, individuals in the D/AO group were older, predominantly women, were less likely to have a conjugal life, had less income and schooling, were more inactive, had lower alcohol intake, had greater frequencies of heart disease, lung disease, depressive symptoms, hypertension and diabetes, had a lower frequency of overweight and had lower grip strength. Lastly, individuals in the D/AO group were younger, had a lower income, greater frequencies of diabetes and obesity and had a higher mean waist circumference compared to the D/NAO group.

The adjusted SHRs obtained in the Fine-Gray model are presented in Table 3. The risk of cardiovascular mortality was significantly higher in individuals with D/AO compared to ND/NAO (SHR 1.85; 95% CI: 1.15–2.97) group independently of age, sex, physical activity level, smoking, alcohol intake, diabetes, hypertension, heart disease, schooling, wealth, marital status and BMI. D/NAO was also associated with cardiovascular mortality (SHR: 1.62; 95% CI: 1.08–2.44), but the effect size of the association was greater in individuals with D/AO. Individuals in the ND/AO group did not present a greater risk of cardiovascular mortality in the present study (Supplementary Table S1).

**Table 3:** Fine-Gray regression model for cardiovascular mortality in 8-year follow-up according to abdominal obesity and dynapenia status in the presence of competing risks, ELSA study (2004/2012)

	Cardiovascular deaths (*n*)	SHR (95% CI)
Non-dynapenic/non-abdominally obese (ND/NAO)	105	1.00
Non-dynapenic/abdominally obese (ND/AO)	142	1.19 (0.86–1.65)
Dynapenic/non-abdominally obese (D/NAO)	37	**1.62 (1.08–2.44)** ^*^
Dynapenic/abdominally obese (D/AO)	38	**1.85 (1.15–2.97)** ^**^

**Note:** SHR: sub-distribution hazard ratio; CI: confidence interval sub-distribution hazard ratios with other causes of mortality as competing events adjusted by age, sex, schooling, total household wealth, marital status, physical activity level, smoking status, alcohol intake, diabetes, hypertension, heart disease and body mass index. ^*^P = 0.019  ^**^P = 0.011.

**Figure 1:**
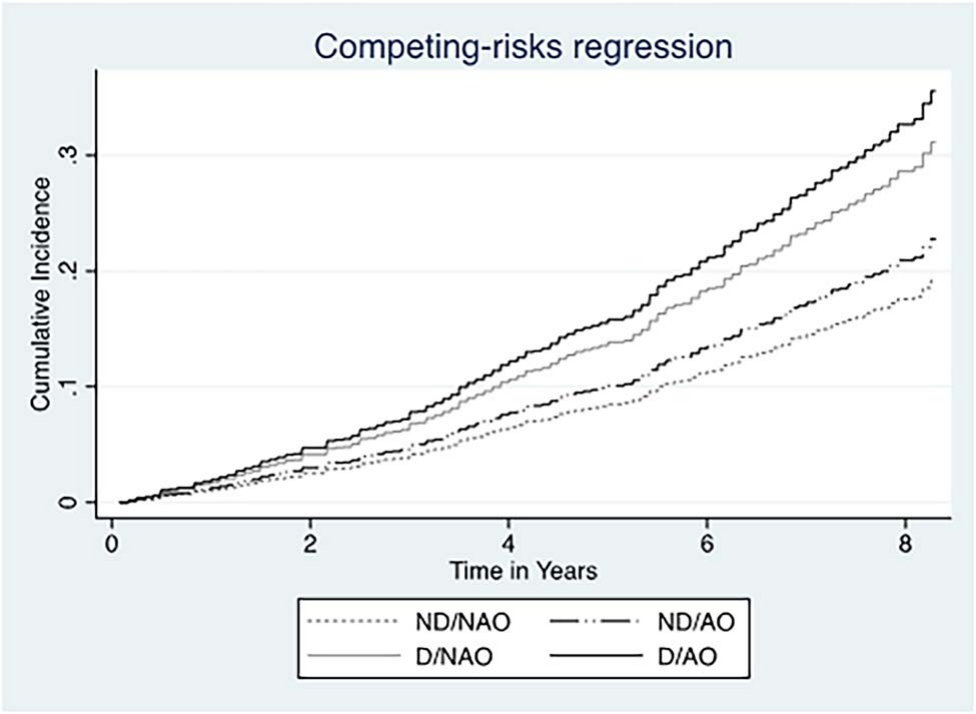
Cumulated incidence function for cardiovascular mortality according to dynapenic abdominal obesity status in presence of competing risks in 7,030 participants of ELSA (2004–2012). ND/NAO: non-dynapenic/non-abdominal obesity; ND/AO: non-dynapenic/abdominal obesity; D/NAO: dynapenic/non-abdominal obesity; D/AO: dynapenic/abdominal obesity. *P* < 0.001.

Figure 1 displays the cumulated incidence functions for cardiovascular mortality according to obesity and dynapenia status in the presence of other causes of death as competing events. Sensitivity analysis excluding individuals younger than 60 years of age and excluding current smokers showed consistent results compared to the principal model (Models 1 and 2). Model 3, excluding participants with heart disease, cancer or stroke at baseline, and Model 4 (without the participants excluded from Models 1, 2 and 3) showed a greater risk of mortality due to cardiovascular disease only for individuals in the D/AO group compared with those in the ND/NAO group (Table 4). In sensitivity Model 5, using BMI, the D/NAO group had an increased risk of cardiovascular mortality compared with the ND/NAO group. Lastly, in a model with dynapenia alone and abdominal obesity alone, only the dynapenic group had a greater risk of cardiovascular mortality compared with non-dynapenic group (SHR: 1.58; 95% CI: 1.19–2.10). The association was not found for the group with abdominal obesity alone (SHR: 1.18; 95% CI: 0.86–1.62).

**Table 4:** Fine-Gray regression models for cardiovascular mortality in the 8-year follow-upaccording to abdominal obesity and dynapenia status in the presence of competing risks, ELSA study (2004/2012)—sensitivity analyses

	Model 1 SHR (95% CI)	Model 2 SHR (95% CI)	Model 3 SHR (95% CI)	Model 4 SHR (95% CI)	Model 5 SHR (95% CI)
	*n* = 4,538	*n* = 6,020	*n* = 4,966	*n* = 2,565	*n* = 7,030
ND/NAO	1.00	1.00	1.00	1.00	1.00
ND/AO	1.18 (0.85–1.65)	1.18 (0.82–1.69)	1.28 (0.79–2.10)	1.34 (0.76–2.35)	1.15 (0.88–1.51)
D/NAO	1.61 (1.07–2.42)^*^	**1.68 (1.07–2.63)^^†^^**	1.35 (0.66–2.77)	1.59 (0.74–3.42)	**1.82 (1.32–2.52)^^¶^^**
D/AO	1.86 (1.14–3.02)^**^	**1.88 (1.13–3.10)^^‡^^**	**3.08 (1.52–6.23)^^§^^**	**3.21 (1.49–6.89)^^||^^**	1.26 (0.75–2.10)

**Note:** SHR: sub-distribution hazard ratio; CI: confidence interval. ND/NAO: non-dynapenic/non-abdominally obese. ND/AO: non-dynapenic/abdominally obese. D/NAO: dynapenic/non-abdominally obese; D/AO: dynapenic/abdominally obese. Model 1: analyses excluding individuals younger than 60 years of age ^*^P = 0.022 ^**^P = 0.012. Model 2: analyses excluding current smokers ^†^*P* = 0.023 ^‡^*P* = 0.014. Model 3: analyses excluding individuals with heart disease, cancer or stroke at baseline ^§^*P* = 0.002. Model 4: analyses excluding individuals younger than 60 years of age, smokers and those with heart disease, cancer or stroke at baseline ^||^*P* = 0.003. Model 5: analyses using BMI ≥ 30 kg/m^2^ to define dynapenic obesity ^¶^*P* < 0.001. Models adjusted by age, sex, schooling, total household wealth, marital status, physical activity level, smoking status, alcohol intake, diabetes and hypertension.

## Discussion

Our key findings showed that individuals in the D/AO group were at a greater risk of mortality due to cardiovascular disease after 8 years of follow-up compared to those in the ND/NAO group. The D/NAO group also had an increased risk of cardiovascular mortality, but the effect size was larger for the participants with the two conditions simultaneously.

Different studies have demonstrated that abdominal obesity and dynapenia are individually associated with heart failure, coronary heart disease, stroke and all-cause death [35–42]. Regarding the relationship between dynapenia and cardiovascular mortality, our findings are similar to those reported in longitudinal studies, such as the Prospective Urban Rural Epidemiology (PURE) study, in which 139,691 individuals from 35 to 70 years of age from 17 countries participated, or an analysis of UK Biobank with 502,293 participants between 40 and 69 years of age and the Ageing and Retirement in Europe (SHARE) study, in which 121,116 individuals older than 50 years of age from 29 countries were analysed. All these studies found an association between dynapenia and the risk of cardiovascular mortality [41, 43, 44].

However, obesity alone was not associated with the risk of cardiovascular disease in the present study. This is a topic that has been widely discussed in the literature and has not yet been resolved. An ‘obesity paradox’ has been described in older people, as overweight is associated with increased risk for cardiovascular diseases but decreased mortality from these diseases [45]. Saad and collaborators analysed data from 12 cohorts to investigate the association between abdominal visceral adipose tissue and all-cause mortality [46]. In four cohorts with a mean age > 65 years, the findings on mortality were inconsistent, as a high abdominal circumference seems to be associated with increased all-cause mortality in individuals ≤65 years, but the association is weaker in older individuals.

Increased adiposity seems to be particularly beneficial for those individuals with low systemic inflammation. This is supported in the literature, which reports that individuals with cardiovascular disease that have high fat mass and low high-sensitivity C-reactive protein have a more favourable prognosis, suggesting that excess total adiposity may not be as detrimental when it is not accompanied by increased systemic inflammation [47].

On the other hand, the results of the present study demonstrate a possible specific synergic mechanism in the combination of abdominal obesity and dynapenia and its effect on cardiovascular mortality. The mechanism that can explain our findings is related to the combined physiopathological action of abdominal obesity and dynapenia, as each condition contributes to and accentuates the other. Obesity generates chronic activation of the immune system, activating macrophages, mastocytes and T lymphocytes and leading to moderate chronic inflammation known as *metaflammation* [48]. This inflammatory state increases the secretion of proinflammatory cytokines (adipokines), such as tumour necrosis factor alpha (TNFα), interleukins 1 and 6 (IL-1 and IL-6) and leptin, which generates insulin resistance, the inhibition of protein synthesis and an increase in muscle catabolism. This increase in cytokines exerts a negative impact on the energy balance, compromises the immune response, blood pressure control, vascular homeostasis, angiogenesis and the metabolism of glucose and lipids, and increases insulin resistance [24].

Regarding musculoskeletal aspects, besides the age-related inhibition of protein synthesis, reduction in type II fibres and reduction in androgen levels needed to stimulate protein synthesis, there is an accumulation of intramuscular fat, denominated myosteatosis [49]. This condition, which is more accentuated in individuals with abdominal obesity, increases oxidative stress and mitochondrial dysfunction in muscle, the consequences of which are lipotoxicity and an increase in reactive oxygen species (ROS), causing insulin resistance and muscle inflammation [24, 50].

Skeletal muscle is an endocrine organ that accounts for around 40% of body mass and participates in metabolic, immune and inflammatory responses, such as blood pressure regulation, the metabolism of insulin, lipids and carbohydrates, as well as the renin-angiotensin system and sympathetic activity [51–56]. In the presence of obesity, therefore, it seems that myocytes also release proinflammatory cytokines (myosins), such as IL-6 and TNFα, in a synergic effect of the activity of myocytes and adipocytes on metabolic dysregulation, aggravating insulin resistance and inflammation [57].

Therefore, dynapenia and dynapenic abdominal obesity may result in greater mitochondrial dysfunction as well as increases in ROS and oxidative stress, impairing the vascular endothelium and cardiomyocytes and leading to atherosclerosis and heart disease [25]. All these factors impair the metabolism and increase the risk of the incidence of cardiovascular diseases and, consequently, the risk of death due to such diseases [21, 23, 51, 52].

The sensitivity model using BMI showed that only the D/NAO group had an increased risk of cardiovascular mortality compared with ND/NAO participants. This could support evidence that central fat is more important to determining health risks associated with obesity in later life [58]. BMI does not reflect the distribution of body fat and does not distinguish between lean mass and fat mass, which is a relevant aspect when estimating cardiovascular risk. Along the same line, as the amount of visceral fat increases with age while muscle mass tends to decrease, this could result in non-significant changes in BMI and the non-perception of increased visceral fat [36].

The sixth sensitivity model, which analysed obesity and dynapenia as isolated conditions, showed that only dynapenia was associated with cardiovascular mortality, which is similar to the result of the main model. However, when dynapenic abdominal obesity was considered, the magnitude of the association was greater. There is evidence that abdominal obesity accelerates the decline in neuromuscular strength [59], which underscores the importance of considering the synergic mechanism of the combination of abdominal obesity and dynapenia and its effect on cardiovascular mortality, as explained above.

The present study has strong points, such as the analysis of a large representative sample of the English population of community-dwelling individuals aged 50 and older and the long follow-up time. Another strength was the use of the Fine-Gray competing-risk model, which enabled the incorporation of mortality risks due to other causes into the analysis, which makes this model more appropriate than the Cox model [30]. Lastly, to the best of our knowledge, this is the first longitudinal study to analyse dynapenic abdominal obesity as a risk factor for cardiovascular mortality in individuals aged 50 and older.

However, the present study has limitations that should be considered. Information on diet quality or changes in eating patterns to enable the adjustment of our models by these factors were not available. Objective methods for the measurement of abdominal obesity, such as DEXA and computed tomography, which provide a much more accurate measurement of body fat were not used. However, these methods require access to sophisticated equipment and qualified technicians and are costly, which limits their implementation in population-based epidemiological studies.

In conclusion, dynapenic abdominal obesity is associated with cardiovascular mortality, with a larger effect size compared to dynapenia alone in individuals older than 50 years of age. Thus, prevention strategies and clinical interventions that enable mitigating the harmful effects of these conditions should be adopted to diminish such risk.

## Supplementary Material

aa-22-1384-File002_afac301

## Data Availability

Data from the English Longitudinal Study of Ageing (ELSA) are available from the UK Data Service for researchers who meet the criteria for access to confidential data under conditions of the End User License http://ukdataservice.ac.uk/media/455131/cd137-enduserlicence.pdf. The data can be accessed from https://www.elsa-project.ac.uk/accessing-elsa-data. Contact with the UK data service regarding access to the English Longitudinal Study of Ageing can be made through the website https://ukdataservice.ac.uk/help/.
