# Supplementary material for: Is dynapenic abdominal obesity a risk factor for cardiovascular mortality? A competing risk analysis
Source: Age Ageing. 2023 Jan 9;52(1):afac301. doi: 10.1093/ageing/afac301 (PMC9831270; doi:10.1093/ageing/afac301)
Supplement: aa-22-1384-File002_afac301 [file aa-22-1384-file002_afac301.docx]

**Is dynapenic abdominal obesity a risk factor for cardiovascular mortality? A competing risk analysis**

**SUPPLEMENTARY MATERIAL**

1. Supplementary Methods
2. Supplemental Figure 1-S. Sample selection flowchart – English Longitudinal Study of Ageing (2004)
3. Supplemental Table 1-S. Fine-Gray final regression model of factors associated with cardiovascular mortality in eight-year follow according to abdominal obesity and dynapenia status in presence of competing risks, ELSA Study (2004/2012).
4. Supplementary References
5. **Supplementary Methods**

**Study population**

ELSA is a panel study that began in 2002 with a representative sample of English adults aged 50 years and older. ELSA recruits participants using multistage, stratified probability sampling with postcode sectors selected in the first stage and household addresses selected in the second stage.

ELSA interviews occur every two years with the administration of questionnaires at the participants’ home. Health examinations, blood collection for the determination of biochemical measures and physical performance tests occur every four years with the visit of a nurse to the participants’ homes. Nurse visits were carried out for the first time in 2004/5 (Wave 2), which corresponded to the baseline of this analysis, as it was the first time that data on obesity and grip strength were collected. Detailed descriptions of the study, sampling procedures and data collection have been published previously [1]. Wave 2 comprised 8,780 participants. For this analysis, only those who were visited by the nurse were eligible (n = 7,666; response rate: 87.3%), 636 of whom were excluded: 336 due to a lack of data on dynapenia, 250 due to a lack of data on waist circumference and 50 due to a lack of data on the covariates used in this study. Therefore, the final analytical sample consisted of 7,030 individuals. We examined all deaths that occurred during the eight-year follow-up (2012/3). This information was available for all participants. The sample selection process is shown in Supplemental Figure 1.

**Cardiovascular mortality**

Information on dates of death and respective causes were obtained from the United Kingdom National Health Service Mortality Registry. Diagnoses for the primary cause of death were recorded using the ICD-10. Codes I00 to I99 were used to classify cardiovascular deaths, codes C00 to C97 were used to classify cancer deaths and codes J00 to J96 were used to classify respiratory deaths. All remaining deaths were classified as ‘other’.

**Dynapenic abdominal obesity**

Abdominal obesity was determined based on waist circumference (WC), which was measured using a flexible metric tape positioned at the midpoint between the last rib and iliac crest with the participant standing, arms alongside the body at the end of the expiratory phase with the abdomen relaxed. Abdominal obesity was defined as WC > 102 cm for men and > 88 cm for women [2].

Muscle strength was determined based on grip strength, which was measured using a *Smedley* handgrip dynamometer (range: 0 to 100 kg) adjusted to the hand size of each participant. The test was performed with the participant standing, arm alongside the trunk and elbow flexed at 90 degrees. Three trials were performed using each hand, with a one-minute rest period between repetitions [3]. The largest strength value for the dominant hand was considered. Dynapenia was defined as grip strength < 26 kg for men and < 16 kg for women, which is the cut-off point recommended by the Foundation for the National Institute of Health Sarcopenia Project (FNIH) as the best indicator of muscle weakness in older adults [4].

**Covariates**

The sociodemographic variables were sex (men = 0; women = 1), age (50-59, 60-69 and 70 or more), marital status (with = 0 or without conjugal life = 1), total household wealth, including financial, housing and physical wealth, such as jewellery and artwork, classified in quintiles [5^th^ quintile (top 20%) = 0; 4^th^ quintile = 1; 3^rd^ quintile = 2; 2^nd^ quintile = 3 and 1^st^ quintile (lowest 20%) = 4] and schooling years (0 to 11 years = 2, 12 to 13 years = 1 and > 13 years = 0), based on the English educational system.

The behavioural characteristics were smoking status (non-smoker = 0, ex-smoker =1 or smoker =2) and weekly frequency of alcohol intake: “never or rarely” (≤ 1 once per week = 0); “frequently” (two to six times per week = 1); “daily” (seven times per week = 2) or “not declared” = 3 [3]. Physical activity level was assessed using three questions from the Physical Activity and Sedentary Behaviour Assessment Questionnaire (PASBAQ) validated by the Health Survey for England [5]. The participants reported the frequency of vigorous (e.g., running/jogging, swimming, cycling, aerobics/gym workout, tennis and digging with a spade), moderate (gardening, cleaning the car, walking at moderate pace, dancing) or light (laundry and home repairs) physical activity using cue cards with different activities to help them interpret different activity intensities. Physical activity was classified as moderate or vigorous = 0 (moderate or vigorous activity at least once per week); low = 1 (only light activity at least once per week); or inactive = 2 (no weekly activity) [6].

Clinical conditions were recorded based on self-reports of a medical diagnosis of systemic arterial hypertension, diabetes, cancer, lung disease, heart disease and stroke (no = 0; yes = 1). Depression was investigated using the 8-item Center for Epidemiological Studies-Depression Scale, for which a score of ≥ 4 was considered indicative of the presence of elevated depressive symptoms (coded 0: < 4 points; coded 1 ≥ 4 points)[7]. Body mass index was estimated using weight in kilograms divided by the square of height in meters (kg/m²). The participants were classified as being in the ideal range (≥ 18.5 and < 25.0 kg/m² = 0), underweight (< 18.5 kg/m² = 1), overweight (≥ 25.0 and < 30.0 kg/m² = 2) or with obesity (≥ 30 kg/m² = 3) [8].

1. **Figure 1-S** Sample selection flowchart – English Longitudinal Study of Ageing (2004).

1. **Table 1-S.** Fine-Gray final regression model of factors associated with cardiovascular mortality in eight-year follow according to abdominal obesity and dynapenia status in presence of competing risks, ELSA Study (2004/2012).

|  | **SHR** | **95% CI** | |
| --- | --- | --- | --- |
| **Abdominal obesity and dynapenia status** |  |  | |
| Non-dynapenic/non-abdominally obese (ND/NAO) | 1.00 | |  |
| Non-dynapenic/abdominally obese (ND/AO) | 1.19 | 0.86 – 1.65 | |
| Dynapenic/non-abdominally obese (D/NAO) | 1.62 | 1.08 – 2.44* | |
| Dynapenic/abdominally obese (D/AO) | 1.85 | 1.15 – 2.97* | |
| **Sociodemographic characteristics** |  |  | |
| Sex (female) | 0.70 | 0.55 – 0.90* | |
| Age, (%) |  |  | |
| 50 -59 | 1.00 | |  |
| 60-69 | 2.80 | 1.43 – 5.48* | |
| 70 or more | 13.1 | 7.05 – 24.52** | |
| Schooling, (%) |  |  | |
| > 13 years | 1.00 | |  |
| 12-13 years | 1.28 | 0.85 – 1.94 | |
| -11 years | 1.43 | 0.99 – 2.06 | |
| Marital status (with conjugal life) | 1.21 | 0.94 – 1.57 | |
| Total household wealth |  |  | |
| Highest quintile | 1.00 |  | |
| 4th quintile | 1.35 | 0.87 – 2.08 | |
| 3rd quintile | 1.23 | 0.78 – 1.95 | |
| 2nd quintile | 1.87 | 1.21 – 2.89* | |
| Lowest quintile | 1.72 | 1.08 – 2.73* | |
| Not applicable | 1.61 | 0.40 – 6.43 | |
| **Behavioural characteristics** |  |  | |
| Smoking |  |  | |
| Non-smoker | 1.00 | |  |
| Ex-smoker | 1.35 | 1.04 – 1.77* | |
| Smoker | 1.35 | 0.94 – 1.97 | |
| Physical activity, (%) |  |  | |
| Moderate/vigorous | 1.00 |  | |
| Low | 1.23 | 0.56 – 2.70 | |
| Inactive | 1.86 | 0.80 – 4.32 | |
| Alcohol intake, (%) |  |  | |
| Never/rarely | 1.00 |  | |
| Frequently | 0.61 | 0.45 – 0.84* | |
| Daily | 0.85 | 0.61 – 1.20 | |
| Not declared | 1.24 | 0.88 – 1.75 | |
| **Clinical conditions** (yes), (%) |  |  | |
| Heart disease | 2.21 | 1.75 – 2.79** | |
| Systemic arterial hypertension | 1.29 | 1.01 – 1.64* | |
| Diabetes | 0.97 | 0.69 – 1.36 | |
| Stroke | 1.41 | 0.98 – 2.03 | |
| **Anthropometry** |  |  | |
| Body mass index, (%) |  |  | |
| Ideal range | 1.00 |  | |
| Underweight | 1.93 | 0.86 – 4.35 | |
| Overweight | 0.82 | 0.59 – 1.11 | |
| Obesity | 0.84 | 0.56 – 1.26 | |

*Note.* SHR: subdistribution hazard ratio; CI: confidence interval, **p* <0.05 ** *p* <0.001.

**4. Supplementary References**

[1] Steptoe A, Breeze E, Banks J, et al. Cohort Profile: The English Longitudinal Study of Ageing. *Int J Epidemiol* 2013; 42: 1640–1648.

[2] National Heart Lung and Blood Institute, National Institutes of Health (NIH) National Heart, Lung, and Blood Institute N. *Clinical Guidelines on the Identification, Evaluation, and Treatment of Overweight and Obesity in Adults*. National Heart, Lung, and Blood Institute, 1998.

[3] Rossi AP, Fantin F, Caliari C, et al. Dynapenic abdominal obesity as predictor of mortality and disability worsening in older adults: A 10-year prospective study. *Clin Nutr* 2016; 35: 199–204.

[4] Alley DE, Shardell MD, Peters KW, et al. Grip strength cutpoints for the identification of clinically relevant weakness. *J Gerontol A Biol Sci Med Sci* 2014; 69: 559–566.

[5] Scholes S, Coombs N, Pedisic Z, et al. Age- and sex-specific criterion validity of the health survey for England Physical Activity and Sedentary Behavior Assessment Questionnaire as compared with accelerometry. *Am J Epidemiol* 2014; 179: 1493–1502.

[6] Shaun Scholes, Jennifer Mindell. Health Survey for England - 2012. Chapter 2: Physical activity in adults. 1–49.

[7] Radloff LS. The CES-D Scale: A Self-Report Depression Scale for Research in the General Population. *Appl Psychol Meas* 1977; 1: 385–401.

[8] Organization WH. *Obesity: Preventing and Managing the Global Epidemic*. World Health Organization, 2000.
